# Supplementary material for: Existence of a continental-scale river system in eastern Tibet during the late Cretaceous–early Palaeogene
Source: Nat Commun. 2021 Dec 13;12:7231. doi: 10.1038/s41467-021-27587-9 (PMC8668954; doi:10.1038/s41467-021-27587-9)
Supplement: Supplementary file 1 — Supplementary Information [file 41467_2021_27587_MOESM1_ESM.pdf]

Supplementary Information

Existence of a continental-scale river system in eastern Tibet during the late Cretaceous–early Palaeogene

Xudong Zhao[1], Huiping Zhang[1,2]*, Ralf Hetzel[3], Eric Kirby[4], Alison R. Duvall[5], Kelin X. Whipple[6], Jianguo Xiong[1], Yifei Li[7], Jianzhang Pang[1], Ying Wang[1], Ping Wang[8], Kang Liu[9], Pengfei Ma[10], Bo Zhang[11], Xuemei Li[1], Jiawei Zhang[1], Peizhen Zhang[2,9]

[1] Lhasa National Geophysical Observation and Research Station, State Key Laboratory of Earthquake Dynamics, Institute of Geology, China Earthquake Administration, Beijing, China

[2] Southern Marine Science and Engineering Guangdong Laboratory (Zhuhai), Zhuhai, China

[3] Institute of Geology and Palaeontology, University of Münster, Münster, Germany

[4] Department of Earth, Marine, and Environmental Sciences, University of North Carolina, Chapel Hill, Chapel Hill, North Carolina, USA

[5] Department of Earth and Space Sciences, University of Washington, Seattle, Washington, USA

[6] School of Earth and Space Exploration, Arizona State University, Tempe, AZ, USA

[7] Key Laboratory of Computational Geodynamics, College of Earth and Planetary Sciences, University of Chinese Academy of Sciences, Beijing, China

[8] School of Geography Science, Nanjing Normal University, Nanjing, China

[9] School of Earth Sciences and Engineering, Sun Yat-Sen University, Guangzhou, China

[10] Department of Earth Science and Engineering, Taiyuan University of Technology, Taiyuan, China

[11] State Key Laboratory of Geological Processes and Mineral Resources, School of Earth Sciences and Resources, China University of Geosciences (Beijing), Beijing, China

Supplementary Information

Supplementary Note

Supplementary Tables1 to 5

Supplementary Figures1 to 8

Supplementary Note

Stratigraphic age and sedimentary characteristics

The late Cretaceous–early Palaeogene deposits in the Sichuan Basin (including Dujiangyan area and Leshan area in this study), Xichang Basin, Huili Basin, and Chuxiong Basin are dominated by fluvial-lacustrine red beds with preserved thicknesses of ~0.5–4 km, displaying highly similar lithology, stratigraphic and sedimentological characteristics. Existing Upper Cretaceous to lower Palaeocene strata have undergone intense modification since the India-Eurasia collision. Our field investigations have revealed that Upper Cretaceous to lower Palaeocene outcrop successions in the studied basins are nearly horizontal and fragmentary, thus magnetostratigraphy method is impracticable for dating these sediments. Moreover, there are too few late Cretaceous or younger detrital zircons to determine the maximum depositional age (MDA) using a weighted mean average age calculation[1]. In this case, palaeontology is a unique way to distinguish the

depositional age of these similar deposits throughout eastern Tibet. We did our best to constrain the age of the sedimentary strata ages using all available palaeontological data. The types of ostracods, charophyta, and few lamellibranchia that are present in the studied sections and basins are well comparable with those from generally accepted late Cretaceous–early Palaeocene sediments in other areas of China (see reviews of refs.[2,3]). Thus, it is convincible to attribute these deposits to late Cretaceous to early Palaeocene time, even if their exact ages are not yet known.

These late Cretaceous–early Palaeogene deposits are consistently characterized by red, thick-bedded, fine- to medium-grained sandstone interbedded with siltstone and mudstone, minor (sandy-) conglomerate layers were restricted to the Dujiangyan area (Supplementary Fig. 1). Large-scale cross bedding, erosional contacts, and upward fining sequences can be observed within lenticular and sheet sandstone bodies, and horizontal-laminations are preserved in these finer deposits. Given the fact that absent evaporates during arid climatic conditions imply existence of significant discharge from river[4]; the paleocurrents are dominated by southward and southeastward[5,6], and tens of meters thick sandstone beds may represent channel deposits, we hypothesize these sediments largely to have deposited in a larger southward flowing river system and associated exorheic lake

and/or floodplain environments. The sedimentology details of each basin are as follows:

Dujiangyan area

In the Dujiangyan area, the late Cretaceous Guankou Formation and early Palaeogene Mingshan Formation are up to 900 m in thickness, and both are mainly characterized by thick-bedded, various grain-size sandstone, thickly to very thickly bedded conglomerates, interbedded with medium to thick-layered siltstone or muddy gypsum layers. The sandstone intervals are decimeter- to several meters-thick and are laterally continuous over tens meters, display tabular or lenticular geometry. Various scales of planar and trough cross-beddings, as well as erosive or sharp basal boundaries can be observed in the conglomerate and sandstone beds. The conglomerate beds are commonly subangular to rounded, and the clasts generally vary in size from 1 to 5 cm in diameter. Massive siltstones and mudstones pinch out laterally, and extent several tens of meters. The major depositional environments of the late Cretaceous and early Palaeogene strata at the Dujiangyan area are thus interpreted as braided river systems. Though palaeontological works in the Dujiangyan area are absent, abundant ostracods such as *Cypridea tera* Su, *Cristocypridea longa* Lee. sp.nov., *Limnocythere morginata* Ho, *"Candona" sinensis* Ho, *Candoniella* sp., *Lineocspris* sp., *Eucypris modica*

Cheu, *Advencypris* sp., *Talicypridea brevis*, *T. chinennsis*, *T. quadrata*, *T. longa, T. desdorte, T. amoena*, *T. baizhanensis*, *Harbinia lanta*, *H. zhengdongensis*, *H. cf. jiadiaensis*, *H. favosa*, *H. jingmenensis*, *H. parafuningesis*, *H. crassa*, *H. oblonga H.postaqunubrata*, *E. angulata*, *E. paomagangensis*, *E. vivata*, *E. debiloides*, *Candona declivis*, *C. huangdianensis, C. extenmata*, *C. maxima, C. dangyangensis*, *C. aurita*, *Candoniella infragilis*, *Obovaticypris hemirofunda* were collected from the corresponding horizon of the Guankou Formation in adjacent area, the age of this set of sediments is thus regarded as late Cretaceous[7–9]. In the same way, the Mingshan Formation was interpreted to be early Palaeogene in age, based on abundant ostracods including *Pinnocyprisalta* Chou, *Limnoc ythere* cf. *bozhenensis* Yang, *Sinocypris funingensis*, S. *elliptica*, *Cyprinotus* sp., *Limnocythere jiansuensis* Yang, *Eucypris loxodeltoides* Wuci, *Cyprois* sp, *Cyprinotus* sp., *Limnocythere exilicosta*, *Paraeucypris privis*[7–9], and charophyta including *Obtusochara*, *Gyrogona*, *Stephanochara*, *Dughiella*, *Charites*, *Croffiella*, *Nenggtichara*, *Peckichara*, *Gobichara*, *Grambastichara*, *Aclistochara*, *Mesochara, Neochara*, *Sphaerochara*, *Latochara*[7].

Leshan area

Conformably overlying the early Cretaceous Jiaguan Formation, the late Cretaceous–early Palaeogene strata in this area contains red- to purple-red colored

sandstone, purple-red siltstone and subordinate mudstone, occasionally interbedded with centimeter-thick gypsum layers. Purple-red mudstones and siltstones are rich in ostracods *Sinocypris funingensis*, *Subulacypris* sp., *Charites sadleri*, *Limnocythere hubeiensis*, *Eucypris levodeitoides*, *Candona* sp., *Cristocypri-dea longa*, *Cypridea (pseudocypridea) gigunten*, *Quadrucypris juvosa, Cypris* sp., *Pinnocypris* sp., *Cyprinotus* sp., *Ilyocypris* sp., *Gyrogona qianjiangica*, charophyta *Crumbutichara longiconia*, and foraminifer *Noniu sichunensis*, indicating an age of late Cretaceous to early Palaeogene[7,10]. The occurrence of massive to horizontal laminated tabular siltstone alternating with tabular mudstone with occasionally medium to strong bioturbation, represents a lacustrine system in arid climatic condition. However, the lack of thick-bedded gypsum layers indicated the lacustrine system in the Leshan area is typical of a hydrologically open, but not playa lake that commonly developed in coeval Jianghan Basin. More importantly, upward-fining successions, flat-beddings, and various scales of cross-beddings can be observed within tabular or lenticular sandstone beds with erosional basal boundaries, and 10–30 cm thick conglomerate can be occasionally observed along the base of the sandstone beds, together indicative of a (sandy) anastomosing or meandering river that developed in regionally low-relief areas.

Xichang Basin

In this study, the stratigraphic and sedimentary characteristics in the Xichang Basin are mainly from previous works[5,7,11–13]. The lithological assemblage is characterized by alternating reddish sandstone, siltstone, and mudstone, as well as meter-thick sandy-conglomerates beds[5,7,11–13]. Specifically, the lower part of the Xiaoba Formation is composed mostly of brick red thick feldspathic quartz sandstone, siliceous–calcareous medium -to- fine-grained sandstone interbedded with lenticular conglomerate beds, and rare mudstone. Sedimentary structures including various-scale cross-stratification, planar cross bedding, horizontal bedding and ripple marks frequently appear in these sandstone beds[12,13]. The sandstone-rich interval also contains gravel interbeds[7,13]. Clasts within the conglomerate layers are rounded and generally sorted, and are commonly imbricated[12], which may represent the deposits of channel fills and scours within a fluvial system. The middle part of the Xiaoba Formation is predominantly by brownish red, dark brownish red mudstone, calcareous siltstone, sandstone, and marl, with occasionally centimetre-to decimeter scale ripple marks horizontal bedding. The upper part of the Xiaoba Formation consists mainly of bright red calcareous siltstone and mudstone, interbedded with medium- to thin bedded calcareous fine-grained sandstone[7,12,13]. Mud cracks, parallel bedding, and small-scale cross bedding are common. Based on these lines of observations, the

Xiaoba Formation was interpreted as the deposits of fluvial and shallow lacustrine environments, as suggested by refs.[7,12,13]. The lower part of the Leidashu Formation is characterized by thickly to very thickly bedded, purplish red to brick red medium- to fine-grained feldspathic quartz sandstone and calcareous siltstone with mudstone interlayers[5,12,13]. The sandstone beds contain cross-bedding and nonerosive basal contacts. The upper part of the Leidashu Formation consists of red and purplish red calcareous quartz siltstone, silty calcareous mudstone and grayish purple fine-grained sandstone with mud crack structures. Sandstone beds with a thickness of ~8 m can be observed at the bottom of this unit[12], which contains large-scale cross bedding and erosional basal boundaries (ref. [12]). The Leidashu Formation was interpreted as fluvial to shallow lacustrine environment in an arid setting based on the ensemble of above observations[12,13]. Coupled with the absence of laterally continuous or thickly bedded evaporites, such fluvial-lacustrine facies association has been interpreted to be deposited in a hydrologically open environment associated with perennial river system[14,15]. Ripples and cross stratification indicate palaeocurrents flowed to the south and southeast[5]. Palaeontological studies suggested the Xiaoba and Leidashu Formations in this area is deposited in the late Cretaceous–early Palaeogene based on abundant freshwater ostracods such as *Cypridea*, *Cypris*, *Darwinula*, *Quadracypris*, *Candona*, *Talicypridea*, *Metacypris*, *Mongilianella*, *Cypridea inclinata*, *C. cuneata*, *C.*

usualis, *C. subquatrata*, *Quadracypris cf. laeta*, *Q. cf. parva*, *Q. quadrata*, *Candona sinensis*, *c. elliputica, c. (M.) xindianensis*, *Candoniella aff. mordvilkoi, Eucypris debiloides, E. angulate, E. virata, Tangxiella* cf. *extrana, Gupris obtusangulla, Clinocypris, Talicyprida, Limnocythere, Mongolocypris, Darwinula* sp. *Aclistcchara* sp. *Lycopterocypris* sp. as well as charophyta including *Charites yangtzensis, Obtusochara prisca, Latochara yuananensis*, *L.guangdongensis*, *Peckichara dangyangensis*, *Charites tenuis*, *Ch. xiaobaensis*, *Ch. guanpingensis*, *Nemegtichara microcylindrica*, *Latochara cylindrical, Croftiella* sp., *Sphaerocara* sp. *O. sphericovalis*, *O. songheensis*, *O. luodianensis*, *C. banyueshanensis*, *Sphaerochara raagha*, *S. parvala*, *Mesochara yanbianensis*, *Latochara*, *Hubeiensis*, *Nodosoclavator puchangheensis*[5,7,11,13].

Huili Basin

In the Huili Basin, the late Cretaceous–early Palaeogene Xiaoba and Leidashu Formations disconformably overlie the Lower Cretaceous Tianmashan Formation. Abundant charophyta such as *Grambastichara communis*, *Charites xiaobaensis*, *Sphae-rochara magna*, *Obtusochara songheensis*, *O. huidongensis*, *O. huiliensis*, and ostracods *Talicypridea*, *Eucypris*, *Cyprois*, *Cypris*, *Paracyprinotus*, *Obtusochara, Mongololianella*, *Spherochara* have been found in the Xiaoba Formation, suggest an age of late Cretaceous[16]. Upward to the Leidashu Formation,

charophyta include *Peckichara zhijiangensis*, *Raskyaechara xinghuaensis*, *Raskyaechara sichuanensis*, *Croftiella leidashuensis*, *Peckichara* sp., *Obtusochara jianglingensis, Gyrogona qianjiangica, Charites sadleri, Grambastichara cf. rudongica*, and ostracods such as *Limnocythere, Candona*, *Darwinula* sp., *Obtusochara*, *Cypris* sp., *Latochara, Croftiella leidashuensis* sp., R*askyaechara xinhuaensis*, *R. sichuanensis* sp. were considered to be evidence of the Palaeocene to early Eocene in age[7,16]. The base of this set of deposits is marked as the obvious appearance of conglomerate to sandy conglomerate layer. This set of strata is up to ~2800 m in thickness within the Zhangguan section, mainly consisting of red and purple-red medium- to fine-grained sandstone, calcareous siltstone and calcareous mudstone, as well as minor sandy conglomerate and coarse-grained sandstone. An ensemble of the thick- to medium-bedded massive siltstone beds and subordinate red mudstone interpreted as exorheic lake and floodplain environments in arid climatic condition, because laterally stable or thickly bedded gypsum intervals are absent. Several meter- to decimeter-thick lenticular and tabular beds with channel scours commonly continue laterally for tens of meters to hundreds of meters. Flat-beddings, trough and planar cross stratification are occasionally observed in the sandstone- to -siltstone beds. Ripples and cross stratification indicate dominant palaeocurrents flowed to the south[17]. We interpret this type of sedimentary assemblage to occur in a large-scale, low-gradient fluvial system, likely

anastomosing or meandering in character, given the widespread development of overbank and floodplain facies.

Chuxiong Basin

In this study, the stratigraphic and sedimentary characteristics of the Jiangdihe and Zhaojiadian Formaitons in the Chuxiong Basin are mainly from previous works[5,18–21]. Ostracods including *Cypridea* cf. *minevensis*, *Darwinula* cf. *leguminella*, *Eucypris* cf. *anluensis*, conchostraca including *Orthestheria daijiatunensis*, *Nemestheria yunannensis*, *Halysestheria* cf. *inflate*, *Aglestheria separate*, and fossil plants *Carpolithus* sp, *Ginkgo* sp, *Phyllotheca* sp, lamellibranchia such as *Trigonioide* sp. cf, T. *laotiae* Hoffet, T. aff. *kobayashi* Hoffet, T. *yunnanensis* Ku et Ma, T. cf. *laoliae* Hoffet were observed from the this set of strata, suggesting that the Jiangdihe and Zhaojiadian Formaitons is also late Cretaceous–early Palaeogene in age[18–20]. Moreover, samples from the Jiangdihe and Zhaojiadian Formations have three late Cretaceous zircon grains (76 Ma, 80 Ma, and 90 Ma). Although the youngest single grain age is not a robust indicator of the true depositional age, it matches the late Cretaceous–early Cenozoic biostratigraphic age of the Jiangdihe and Zhaojiadian Formations.

The late Cretaceous Jiangdihe Formation across most of the Chuxiong Basin is mainly composed of red to gray-purple feldspathic sandstone, siltstone, and

mudstone, interbedded with pebbly sandstones to conglomerate, which has commonly been interpreted as fluvial and shallow lacustrine facies[5,18–21]. In particular, a very thickly bedded wedge-shaped sandstone body, known as "Fangjiahe wedge-sandstone body", has been observed from the lower part of the Jiangdihe Formation in southern basin[19]. This sandstone body is characterized by grey purple thick sandstone interbedded with purplish red siltstone and mudstone, which are laterally continuous over scales of hundreds of meters and about 4.3 km longitudinally[19]. Individual sandstone set with various-scale cross stratifications is commonly >2 m thick[19], which was likely be deposited in a large-scale fluvial system[22]. The lithological and facies assemblages of the Leidashu Formation are similar to those of the Jiangdihe Formation. Although this unit in the Yijiu section has been interpreted as deposits of dominated shallow-to-marginal lacustrine system by ref.[5], slightly synchronous lateral facies changes may exist across the basin[19,20]. The Zhaojiadian Formation in the Guatang-Sanzhi section (near the Yijiu section) is characterized by purplish red sandstone interbedded with siltstone and mudstone beds[20]. Various-scale cross stratifications and ripple marks can be observed in individual sandstone beds with basal granule lags[20,22]. Mud cracks, calcareous nodules, and bioturbation structures are common in finer-grained deposits, which are considered to be typical features of floodplain or overbank depositional environment (refs.[20,22]). These lines of evidence suggest that the

Zhaojiadian Formation in the Guotang-Sanzhi section was mainly deposited in a fluvial environment[20]. From a basin-scale perspective, such fluvial-lacustrine facies associations were thought to be major diagnostic criterion of overfilled lake basin that was defined as the rate of supply of sediment + water consistently exceeds potential accommodation[14,15]. Generally, this type of lake-basin is very closely related to perennial river systems. Paleocurrent measurements of the Jiangdihe and Zhaojiadian Formations show southwestward and southeastward palaeocurrents[5,21]. The biofacies records in the Chuxiong Basin provide further evidence. It shows that the fossil is dominated by ostracodes, freshwater lamellibranchia, fish species, and plant fossils which are typical of the paleontological assemblages in many other hydrologically open lakes (refs.[14,15]).

Supplementary Table 1. Kolmogorov–Smirnov (K-S) test *D* values and Kuiper test *V* values of the isotopic ages of the detrital zircons from K_2–E_1 strata, potential source areas and modern river samples. The results of this analysis are generated by the DZStats software[23]. Green fillings indicate an increase in similarity whereas red fillings show a decrease in similarity.

Dzstats Results	Samples		Potential Source Areas								Modern River sands			Late Cretaceous–Early Paleogene sediments						
			SP-GZ	YIDUN	UYZ	INDO	QL	W-SC	S-QT	N-QT	MJ-R	JS-R	YL-R	DJ	LS	XC	HL	CX	SM
K-S test D statistic	Potential Source Areas	SP-GZ	0.000	0.169	0.212	0.482	0.284	0.387	0.283	0.273	0.145	0.113	0.252	0.114	0.142	0.145	0.099	0.083	0.082
		YIDUN	0.169	0.000	0.232	0.610	0.329	0.265	0.164	0.328	0.256	0.191	0.400	0.100	0.211	0.195	0.136	0.204	0.135
		UYZ	0.212	0.232	0.000	0.439	0.175	0.389	0.303	0.287	0.173	0.132	0.248	0.229	0.107	0.090	0.138	0.147	0.148
		INDO	0.482	0.610	0.439	0.000	0.311	0.821	0.735	0.720	0.578	0.522	0.560	0.586	0.453	0.454	0.526	0.460	0.507
		QL	0.284	0.329	0.175	0.311	0.000	0.549	0.471	0.457	0.299	0.236	0.299	0.327	0.179	0.180	0.223	0.234	0.224
		W-SC	0.387	0.265	0.389	0.821	0.549	0.000	0.246	0.420	0.398	0.384	0.550	0.325	0.432	0.418	0.368	0.437	0.384
		S-QT	0.283	0.164	0.303	0.735	0.471	0.246	0.000	0.223	0.248	0.243	0.393	0.224	0.302	0.319	0.267	0.318	0.270
		N-QT	0.273	0.328	0.287	0.720	0.457	0.420	0.223	0.000	0.244	0.232	0.389	0.256	0.287	0.306	0.251	0.305	0.256
	Modern River sands	MJ-R	0.145	0.256	0.173	0.578	0.299	0.398	0.248	0.244	0.000	0.144	0.278	0.193	0.173	0.168	0.137	0.189	0.164
		JS-R	0.113	0.191	0.132	0.522	0.236	0.384	0.243	0.232	0.144	0.000	0.260	0.118	0.091	0.089	0.085	0.091	0.096
		YL-R	0.252	0.400	0.248	0.560	0.299	0.550	0.393	0.389	0.278	0.260	0.000	0.337	0.214	0.209	0.284	0.206	0.309
	Late Cretaceous –Early Paleogene sediments	DJ	0.114	0.100	0.229	0.586	0.327	0.325	0.224	0.256	0.193	0.118	0.337	0.000	0.172	0.179	0.123	0.150	0.122
		LS	0.142	0.211	0.107	0.453	0.179	0.432	0.302	0.287	0.173	0.091	0.214	0.172	0.000	0.052	0.098	0.080	0.107
		XC	0.145	0.195	0.090	0.454	0.180	0.418	0.319	0.306	0.168	0.089	0.209	0.179	0.052	0.000	0.081	0.079	0.104
		HL	0.099	0.136	0.138	0.526	0.223	0.368	0.267	0.251	0.137	0.085	0.284	0.123	0.098	0.081	0.000	0.083	0.059
		CX	0.083	0.204	0.147	0.460	0.234	0.437	0.318	0.305	0.189	0.091	0.206	0.150	0.080	0.079	0.083	0.000	0.121
		SM	0.082	0.135	0.148	0.507	0.224	0.384	0.270	0.256	0.164	0.096	0.309	0.122	0.107	0.104	0.059	0.121	0.000
Kuiper test V statistic	Potential Source Areas	SP-GZ	0.000	0.173	0.230	0.485	0.332	0.387	0.291	0.474	0.273	0.166	0.368	0.136	0.146	0.178	0.151	0.094	0.148
		YIDUN	0.173	0.000	0.233	0.610	0.358	0.311	0.294	0.476	0.280	0.194	0.401	0.116	0.212	0.217	0.145	0.210	0.153
		UYZ	0.230	0.233	0.000	0.546	0.238	0.389	0.310	0.487	0.290	0.185	0.396	0.231	0.174	0.141	0.151	0.221	0.157
		INDO	0.485	0.610	0.546	0.000	0.494	0.821	0.735	0.720	0.581	0.553	0.567	0.587	0.460	0.476	0.529	0.460	0.507
		QL	0.332	0.358	0.238	0.494	0.000	0.554	0.471	0.631	0.403	0.301	0.522	0.359	0.258	0.213	0.274	0.301	0.266
		W-SC	0.387	0.311	0.389	0.821	0.554	0.000	0.275	0.428	0.398	0.386	0.550	0.327	0.432	0.422	0.368	0.442	0.391
		S-QT	0.291	0.294	0.310	0.735	0.471	0.275	0.000	0.271	0.252	0.243	0.393	0.271	0.302	0.320	0.296	0.321	0.305
		N-QT	0.474	0.476	0.487	0.720	0.631	0.428	0.271	0.000	0.351	0.397	0.389	0.468	0.445	0.464	0.456	0.450	0.481
	Modern River sands	MJ-R	0.273	0.280	0.290	0.581	0.403	0.398	0.252	0.351	0.000	0.259	0.287	0.277	0.252	0.273	0.261	0.281	0.304
		JS-R	0.166	0.194	0.185	0.553	0.301	0.386	0.243	0.397	0.259	0.000	0.370	0.122	0.134	0.148	0.115	0.143	0.135
		YL-R	0.368	0.401	0.396	0.567	0.522	0.550	0.393	0.389	0.287	0.370	0.000	0.393	0.346	0.359	0.380	0.361	0.424
	Late Cretaceous –Early Paleogene sediments	DJ	0.136	0.116	0.231	0.587	0.359	0.327	0.271	0.468	0.277	0.122	0.393	0.000	0.172	0.195	0.125	0.157	0.139
		LS	0.146	0.212	0.174	0.460	0.258	0.432	0.302	0.445	0.252	0.134	0.346	0.172	0.000	0.077	0.110	0.117	0.110
		XC	0.178	0.217	0.141	0.476	0.213	0.422	0.320	0.464	0.273	0.148	0.359	0.195	0.077	0.000	0.115	0.122	0.137
		HL	0.151	0.145	0.151	0.529	0.274	0.368	0.296	0.456	0.261	0.115	0.380	0.125	0.110	0.115	0.000	0.118	0.098
		CX	0.094	0.210	0.221	0.460	0.301	0.442	0.321	0.450	0.281	0.143	0.361	0.157	0.117	0.122	0.118	0.000	0.141
		SM	0.148	0.153	0.157	0.507	0.266	0.391	0.305	0.481	0.304	0.135	0.424	0.139	0.110	0.137	0.098	0.141	0.000

Note: SP-GZ = Songpan-Gnazi Terrane; UYZ= Upper Yangtze Terrane; Indo = Indochina; QL = Qinling Belt; W-SC =Western South China Block; S-QT = Southern Qiangtang Terrane; N-QT: Northern Qiangtang Terrane; MJ-R = Minjiang River; JS-R = Jinsha River; YL-R = Yalong River; DJ = Dujiangyan area; LS = Leshan area; XC = Xichang Basin; HL = Huili Basin; CX = Chuxiong Basin; SM = Simao Basin.

Supplementary Table 2. Parameters used in the landscape evolution model.

Parameter	Value
Drainage area	2000 × 1200 km
Diffusion coefficient K_d	0.01 m^2 yr^{-1}
Uplift rate	4 × 10^{-5} m yr^{-1}
River incision coefficient K_s	1.11 × 10^{-6} $m^{0.1}$ yr^{-1}
m	0.45
n	1

Supplementary Table 3. Sampling locations and descriptions in this study

Station	Sample ID	Latitude and Longitude	Sampling horizon	Heavy mineral	Detrital zircon	Petrography
Dujiangyan	CX-01	N30°57′10.69″ E103°33′24.37″	Guankou Fm.			√
Dujiangyan	CX-02	N30°55′37.40″ E103°34′25.23″	Mingshan Fm.	√	√	√
Dujiangyan	CX-03	N30°55′35.09″ E103 °34′20.38″	Guankou Fm.	√	√	√
Dujiangyan	CX-04	N30°55′23.94″ E103°33′53.11″	Guankou Fm.			√
Dujiangyan	CX-05	N30°55′36.35″ E103 °33′33.26″	Guankou Fm.	√		
Dujiangyan	CX-06	N30°58′43.57″ E103°34′37.20″	Mingshan Fm.			√
Dujiangyan	CX-07	N30°41′34.82″ E103°29′4.49″	Mingshan Fm.	√	√	√
Dujiangyan	CX-08	N30°41′46.53″ E103°28′57.76″	Mingshan Fm.			√
Leshan	CX-13	N29°39′29.66″ E103°40′7.74″	Mingshan Fm.	√		
Leshan	CX-14	N29°39′49.00″ E103°39′45.70″	Mingshan Fm.	√	√	√
Leshan	CX-15	N29°42′20.20″ E103°40′34.51″	Mingshan Fm.	√		
Leshan	CX-17	N29°35′51.34″ E103°27′39.71″	Guankou Fm.	√		√
Leshan	CX-18	N29°35′51.34″ E103°27′39.71″	Mingshan Fm.	√		√
Leshan	CX-19	N29°35′57.86″ E103°27′34.42″	Mingshan Fm.	√	√	√
Leshan	CX-20	N29°35′46.53″ E103°27′44.67″	Mingshan Fm.	√		√
Leshan	CX-21	N29°35′46.95″ E103°27′44.12″	Mingshan Fm.	√		√
Leshan	CX-22	N29°35′40.63″ E103°27′46.80″	Guankou Fm.	√		√
Xichang	CX-23	N28°1′59.94″ E102°28′42.46″	Leidashu Fm.	√	√	√
Xichang	CX-24	N28°6′11.65″ E102°30′47.54″	Leidashu Fm.	√	√	√
Xichang	CX-25	N28°7′30.01″ E102°30′44.20″	Xiaoba Fm.	√	√	√
Huili	CX-29	N26°25′14.80″ E102°20′26.51″	Leidashu Fm.			√
Huili	CX-30	N26°27′18.81″ E102°20′32.57″	Leidashu Fm.	√	√	
Huili	CX-31	N26°31′9.11″ E102°19′13.53″	Leidashu Fm.	√	√	
Huili	CX-32	N26°31′23.12″ E102°18′21.74″	Xiaoba Fm.	√	√	√
Chuxiong	CX-33	N25°53′15.94″ E101°42′21.43″	Jiangdihe Fm.			√
Chuxiong	CX-34	N25°54′18.54″ E101°41′46.08″	Zhaojiadian Fm.	√	√	√
Chuxiong	CX-35	N25°55′8.63″ E101°43′34.54″	Jiangdihe Fm.	√	√	√
Chuxiong	CX-36	N25°55′28.46″ E101°43′3.38″	Jiangdihe Fm.	√	√	

Note: "√" represent available provenance data of the samples analyzed

Supplementary Table 4. Bulk petrography composition counts for samples in this study.

Sample	Qm	Qp	F	Lv	Ls	Lc	Lm	Hb	M	Total
CX-01	240	41	6	0	40	18	69	0	13	427
CX-02	178	43	4	0	29	42	86	0	9	391
CX-03	150	16	2	0	0	249	136	0	22	575
CX-04	127	57	2	0	43	31	125	0	10	395
CX-06	283	35	9	1	24	0	81	0	53	486
CX-07	255	39	7	0	24	117	39	0	4	479
CX-08	260	39	7	0	24	0	78	0	12	420
CX-14	356	24	48	0	12	0	13	0	0	453
CX-17	402	25	47	0	2	56	3	0	3	538
CX-18	347	21	25	0	0	17	3	0	2	416
CX-19	334	11	48	0	3	0	2	0	47	445
CX-20	354	26	41	0	8	0	11	0	1	441
CX-21	380	29	39	0	13	0	3	0	2	466
CX-22	344	6	49	0	64	65	2	0	20	550
CX-23	428	14	48	0	1	97	8	0	5	601
CX-24	257	35	29	0	0	107	17	0	5	450
CX-25	248	10	47	0	0	140	16	0	20	481
CX-29	290	21	21	1	10	60	13	0	0	416
CX-32	323	30	36	1	0	196	26	0	4	616
CX-33	382	26	49	0	4	0	37	0	3	501
CX-34	229	34	15	0	3	152	21	0	3	457
CX-35	365	26	42	1	0	0	43	0	4	481

Qm–Single crystal quartz; Qp–Polycrystalline; F–feldspar; L–lithic, Lv–volcanic lithic; Ls–terrestrial sedimentary lithic; Lc–carbonate lithic; Lm– metamorphic lithic; Hb –Hornblende and pyroxene; M–Single crystal layered silicate detritus.

Supplementary Table 5. Heavy mineral counts for samples in this study.

Samples	Zircon	Apatite	Rutile	Sphene	Leucoxene	Garnet	Tourmaline	Anatase	Ilmenite	Hematite	Magnetite	Others
CX-02	14%	0%	4%	0%	11%	0%	0%	0%	8%	27%	28%	6%
CX-03	18%	1%	4%	4%	9%	0%	0%	0%	9%	22%	28%	4%
CX-05	19%	1%	4%	0%	10%	9%	0%	0%	4%	20%	22%	10%
CX-07	15%	0%	4%	0%	7%	0%	1%	1%	7%	31%	15%	17%
CX-13	17%	0%	4%	0%	13%	6%	0%	0%	0%	30%	27%	2%
CX-14	18%	0%	4%	0%	11%	1%	2%	0%	2%	34%	26%	3%
CX-15	11%	2%	5%	1%	8%	4%	3%	0%	3%	30%	26%	7%
CX-17	20%	0%	4%	0%	7%	4%	1%	1%	7%	23%	26%	5%
CX-18	14%	0%	5%	2%	10%	4%	1%	2%	4%	28%	24%	5%
CX-19	20%	0%	4%	0%	7%	2%	1%	0%	1%	33%	26%	4%
CX-20	21%	0%	4%	0%	7%	1%	1%	0%	1%	32%	29%	4%
CX-21	19%	0%	4%	0%	5%	1%	2%	0%	0%	39%	26%	4%
CX-22	17%	0%	3%	0%	4%	3%	1%	1%	4%	35%	24%	8%
CX-23	19%	1%	3%	0%	8%	0%	1%	1%	0%	33%	29%	6%
CX-24	17%	1%	4%	0%	6%	0%	0%	1%	0%	38%	29%	4%
CX-25	17%	4%	2%	0%	5%	0%	1%	1%	0%	34%	19%	15%
CX-30	17%	1%	3%	0%	10%	2%	1%	1%	0%	32%	27%	5%
CX-31	15%	3%	3%	0%	5%	1%	2%	2%	0%	35%	30%	5%
CX-32	13%	1%	2%	0%	7%	0%	1%	4%	0%	41%	22%	9%
CX-34	19%	1%	2%	0%	7%	0%	1%	2%	0%	36%	26%	6%
CX-35	21%	0%	2%	0%	4%	0%	0%	2%	0%	42%	25%	4%
CX-36	7%	1%	0%	0%	1%	1%	1%	2%	0%	40%	13%	35%

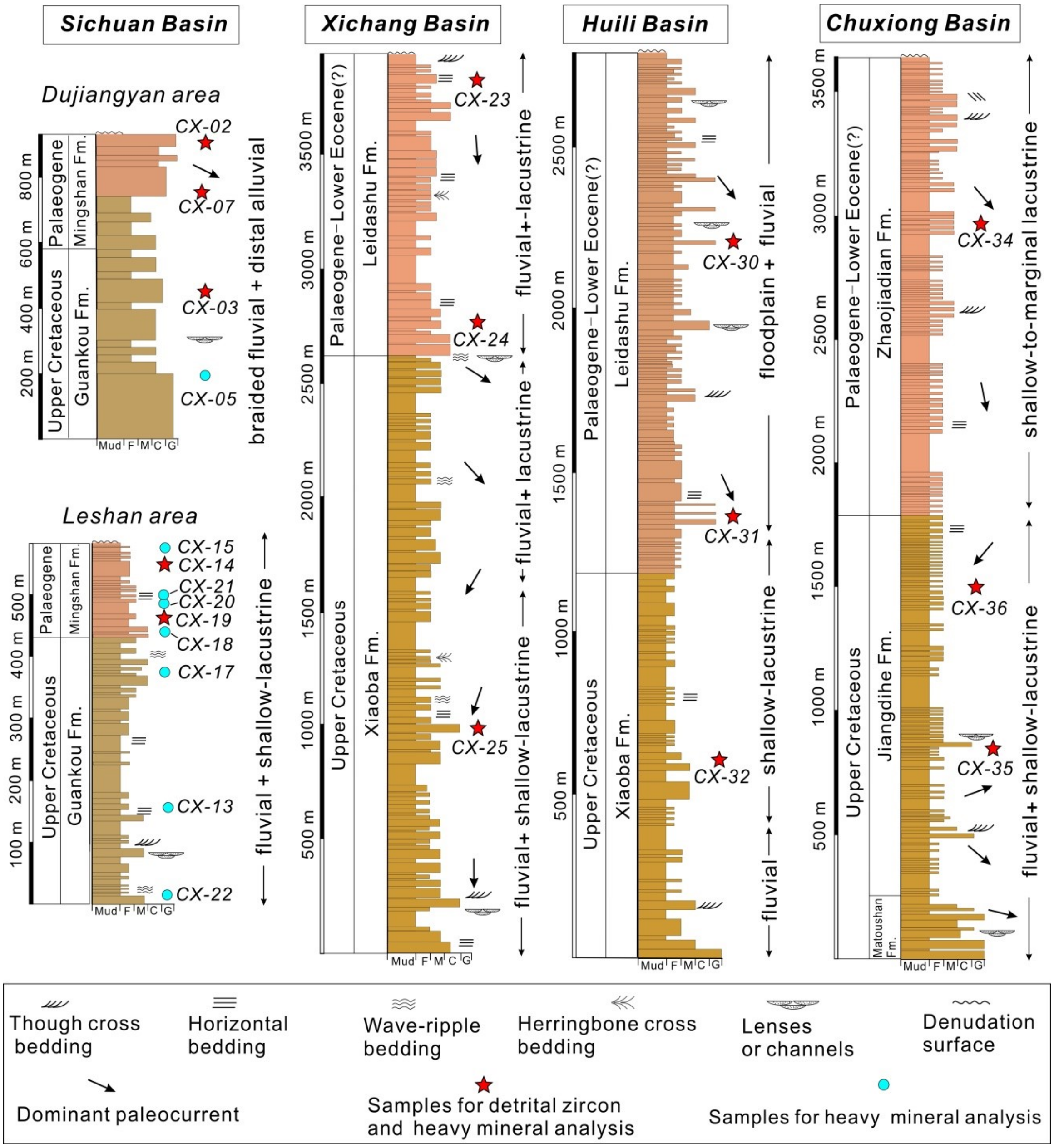

Supplementary Figure 1. Stratigraphic columns of the K_2–E_1 successions. Palaeocurrent directions are from previous studies[5,6] and our own field observations. Stratigraphic data from the Xichang and Chuxiong Basins are from ref.[5]. For detailed lithological characteristics and sedimentary facies interpretations see the Supplementary Note.

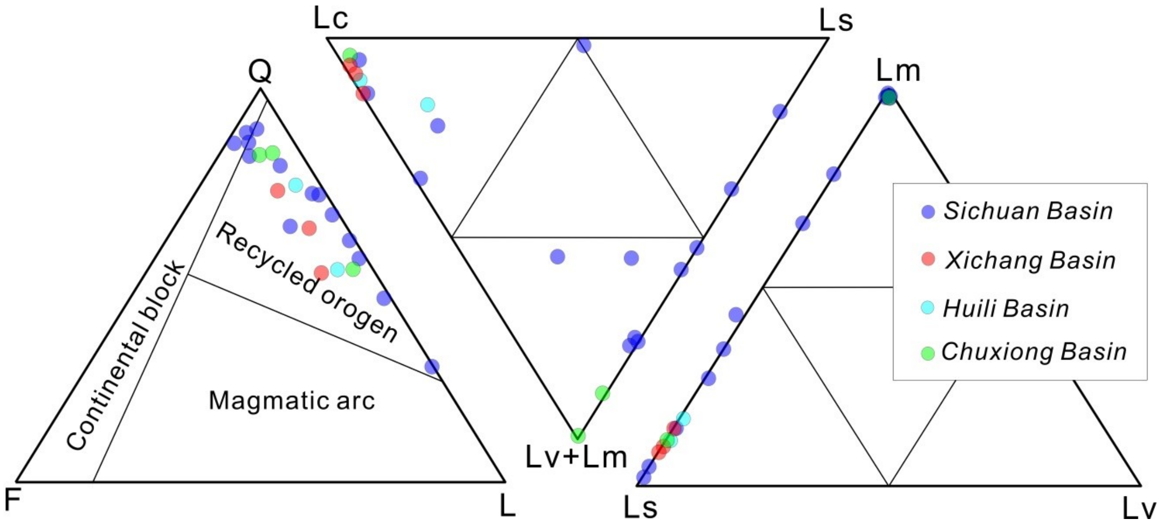

Supplementary Figure 2. Ternary diagrams summarizing the K_2–E_1 sandstone petrographic data. Provenance fields after refs.[24,25]. Q–quartz, F–feldspar, L–lithics, Lv–volcanic lithic; Ls–terrestrial sedimentary lithic; Lc–carbonate lithic; Lm–metamorphic lithic.

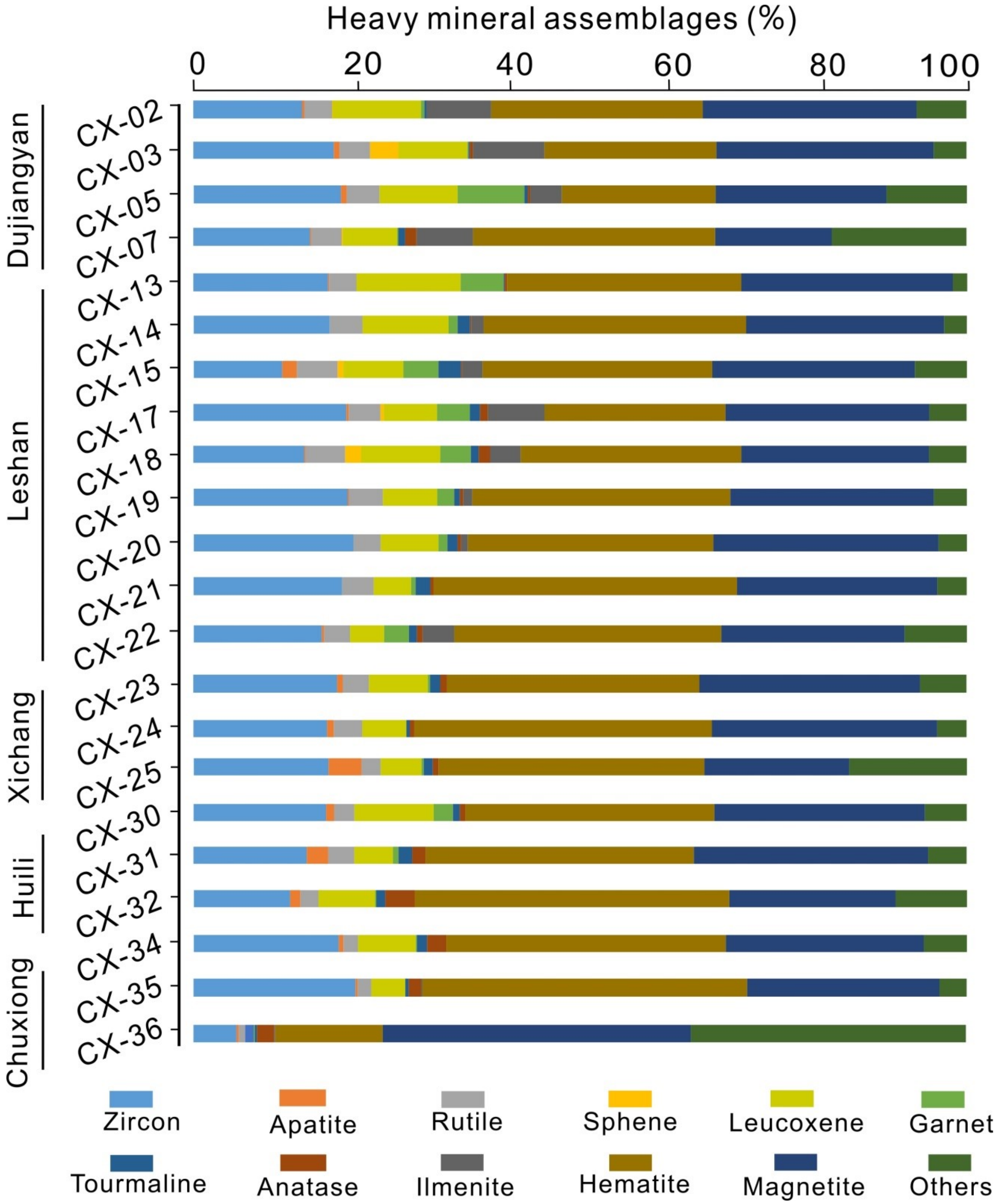

Supplementary Figure 3. Heavy mineral assemblages of the K_2–E_1 sediments. Basin or section abbreviations as in Fig. 1. Sampling locations are shown in Supplementary Fig. 1 and Supplementary Table 3.

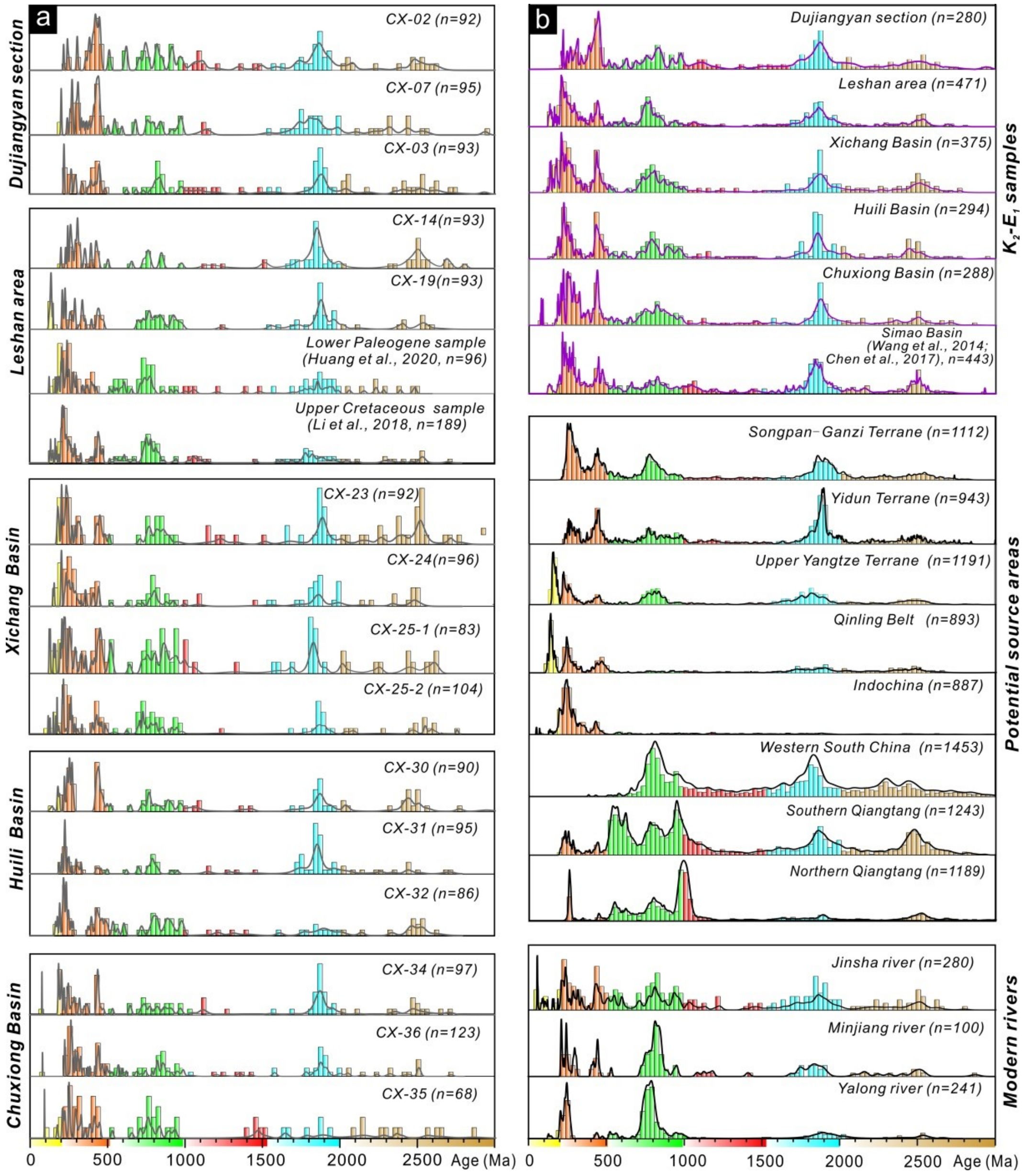

Supplementary Figure 4. Zircon U-Pb data of Cretaceous–early Palaeogene strata and potential source regions. a, Age distributions for Cretaceous–early Palaeogene samples from the different basins (areas). *n*—number of concordant ages. **b,** Compilation of data from studied basins, potential source areas, and modern river samples. Black, grey, and purple lines are normalized probability density functions. Zircon U-Pb data of late Cretaceous–early Palaeogene samples are from this study and were compiled from refs.[6,26-28]. Data of potential source areas and modern river samples are from refs.[6,26] and references therein.

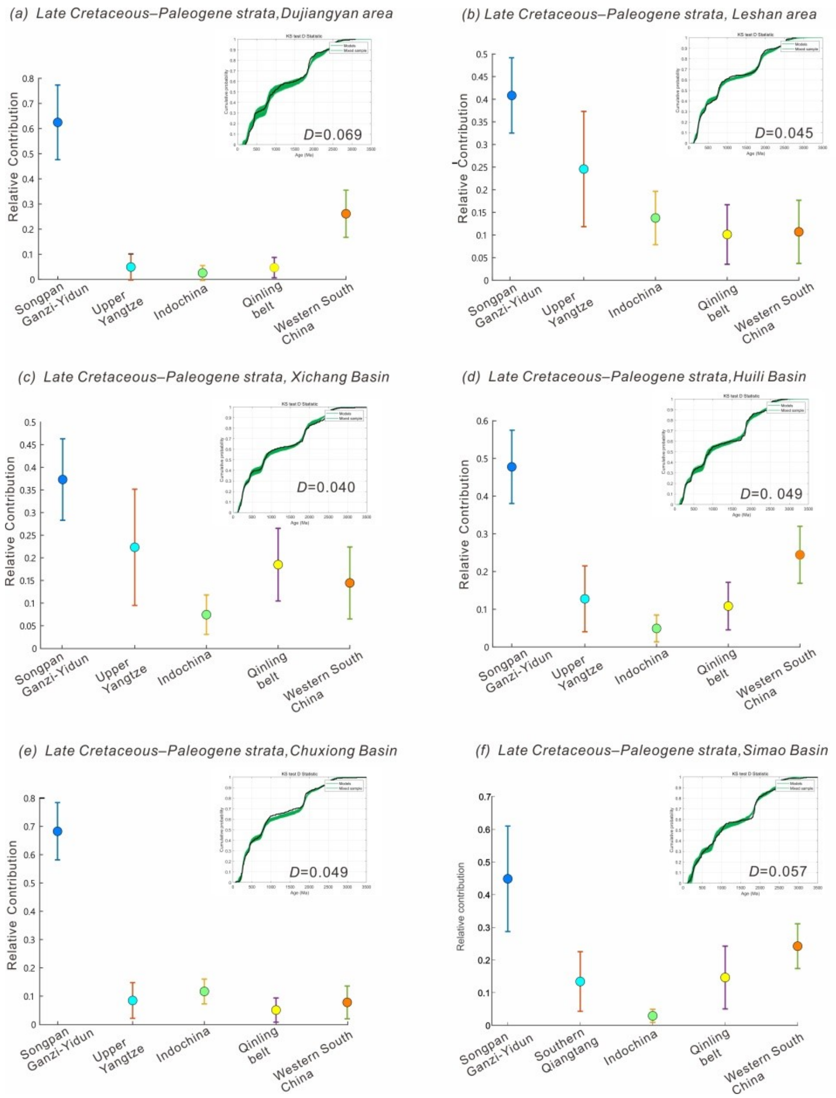

Supplementary Figure 5. Plot of modelled relative contributions from potential source areas. Panels show DZmix results for the Late Cretaceous–Paleogene strata from **a** Dujiangyan, **b** Leshan, **c** Xichang, **d** Huili, **e** Chuxiong, and **f** Simao areas. Cumulative frequency plots of the observed U-Pb data for K_2–E_1 samples and best fits using the Kolmogorov–Smirnov (*K-S*) test statistic (D), generated by the Monte Carlo unmixing model[29]. Model results show that the Songpan-Ganzi and Yidun terranes are principal source areas for all K_2–E_1 basins. The Upper Yangtze terrane provides a non-negligible source for sediment in the Leshan area and Xichang basin.

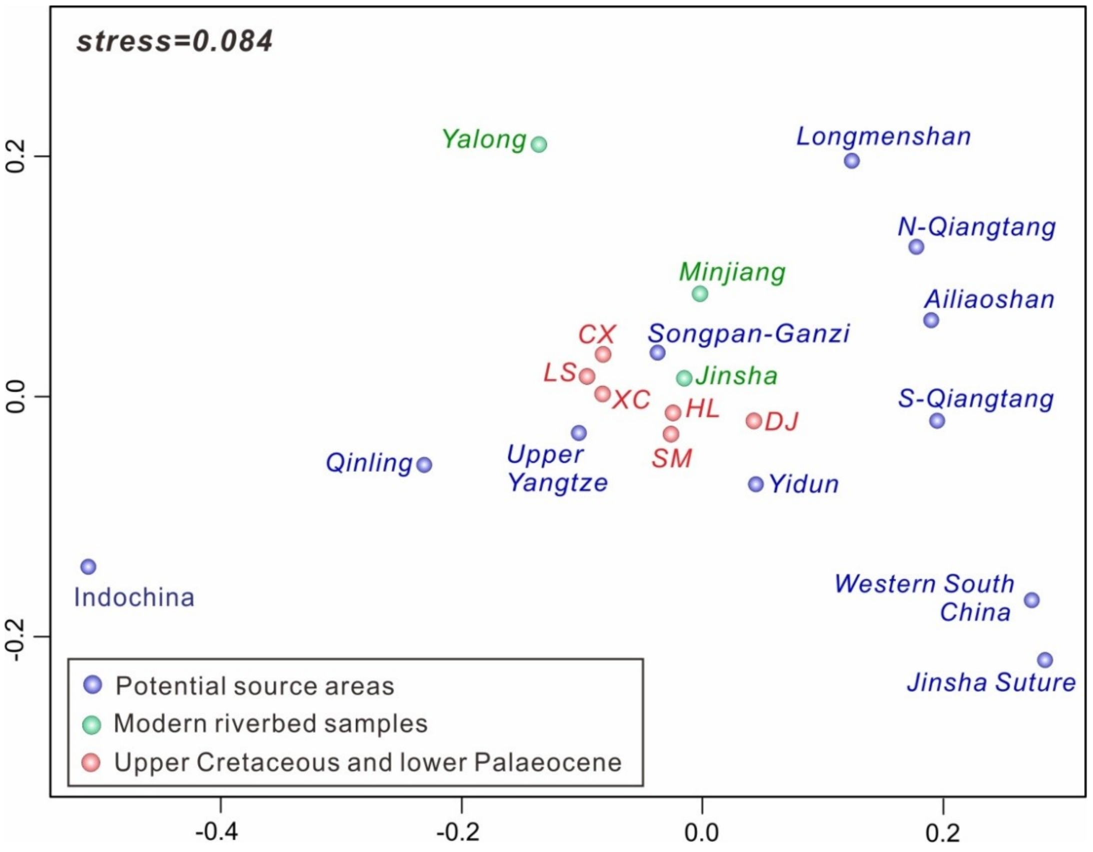

Supplementary Figure. 6. Multidimensional scaling plot of age datasets from K_2–E_1 strata, potential source areas, and modern river samples. Different types of zircon U-Pb samples are represented by circles of different colour. The closer two samples (circles) are, the greater is the similarity between their age distributions. Multidimensional scaling plots show that zircon age populations from all late Cretaceous–early Palaeogene samples from the Sichuan, Xichang, Huili, and Chuxiong basins are remarkably similar to those from the Triassic flysch in the Songpan-Ganzi–Yidun terranes and the pre-late Cretaceous strata in the Upper Yangtze terrane, as well as those from modern river sand from the Minjiang and Jinsha rivers. The stress values of the scaling-plot statistics indicate a good to fair goodness of fit[30].

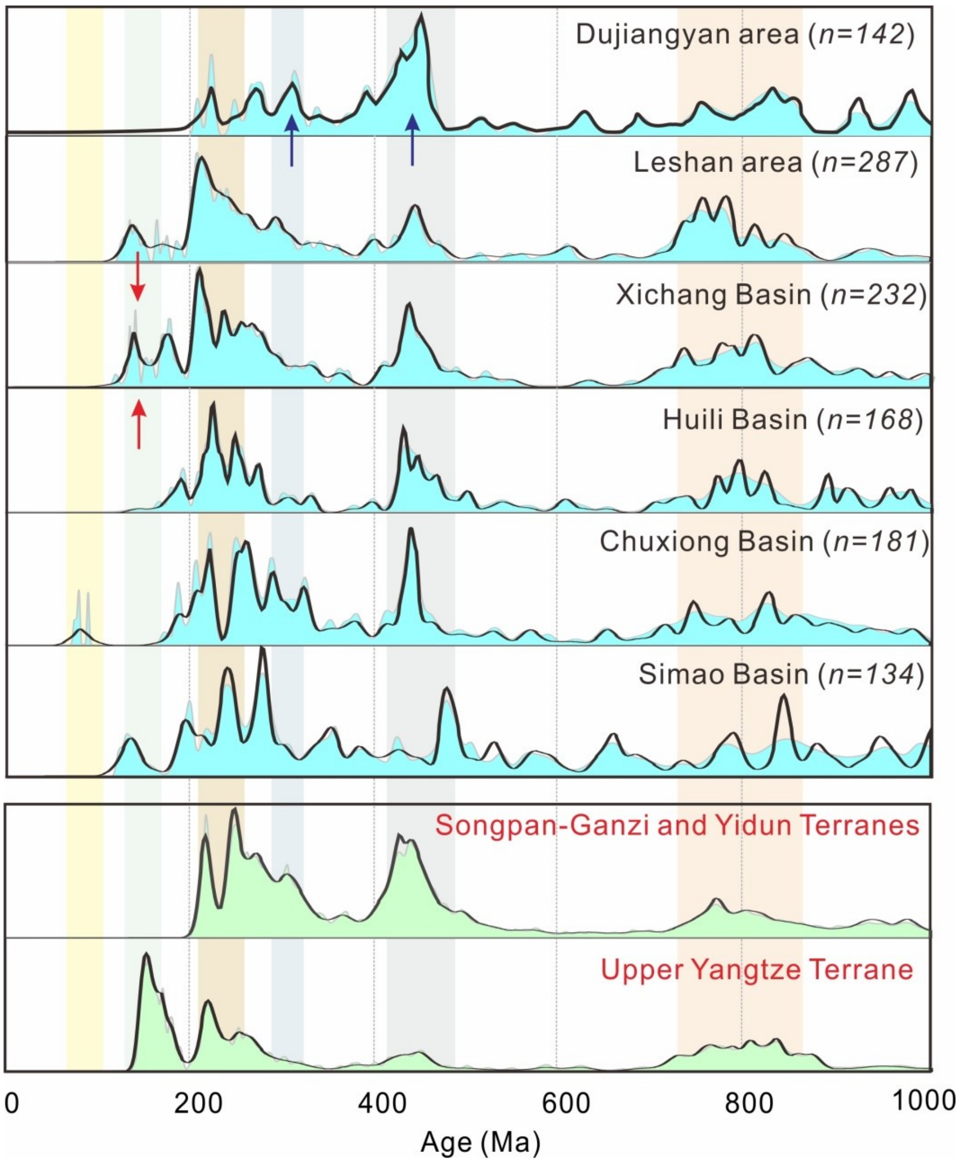

Supplementary Figure 7. Comparison of normalized probability density functions (shaded areas) and kernel density plots (black curves) of zircon U-Pb ages (＜1000 Ma) between the K_2–E_1 samples and those from the Songpan-Ganzi and Yidun terranes, and the Upper Yangtze terrane. Red arrows indicate increase or decrease of the＜200 Ma age population. Blue arrows indicate increase of the ~310 Ma and ~450 Ma age peaks.

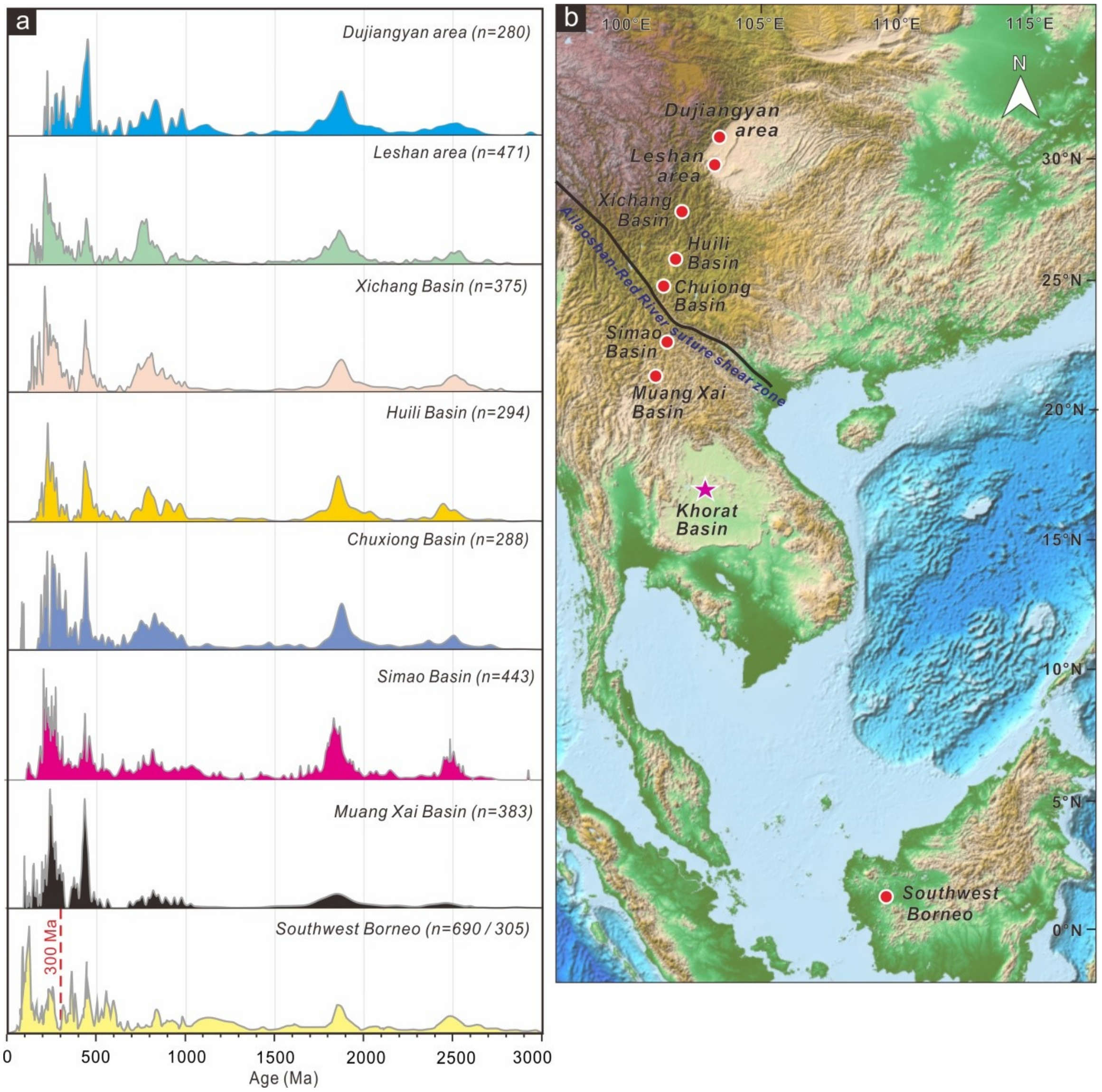

Supplementary Figure 8. Comparison of detrital zircon U-Pb results from late Cretaceous and/or early Palaeogene deposits at the eastern margin of the Tibetan Plateau and areas to the south. **a**, Probability density function plots of compiled detrital zircon U-Pb data. Consistency in multiple age peaks of K_2–E_1 deposits from these basins suggests existence of a through-going river system that existed during K_2–E_1. *n*—number of grains. Please note that n = 690 / 305 in the plot of the southwest Borneo means the number of zircon ages in the range of 0–300 Ma is 690, and the number of zircon ages in

the range of 300–3000 Ma is 305, because a dominance of Mesozoic detrital zircons would inhibit efficient plotting and hinder effective comparison with other samples. **b**, Topographic map showing basin localities. Detrital zircon data were compiled from this study, and refs.[6,26–28,31–33]. The base map of (**b**) was taken from open-access https://www.ngdc.noaa.gov/mgg/global/.

Supplementary References

1. Sharman and Malkowski. Needles in a haystack: Detrital zircon U-Pb ages and the maximum depositional age of modern global sediment. *Earth Sci Rev*. **203**, 103109 (2020).
2. Wang, Y. Q., Li, Q., Bai, B., Jin, X., Mao, F. Y., Meng, J. Paleogene integrative stratigraphy and timescale of China. *Sci China Earth Sci.* **62**, 287–309 (2019).
3. Xi, D. P., Wan, X. Q., Li, G. B., Li, G. Cretaceous integrative stratigraphy and timescale of China. *Sci China Earth Sci.* **62**, 256–286 (2019).
4. Zheng, H. B., Clift, P. D., Wang, P., Tada, R., Jia, J., He, M. & Jourdan, F. Pre-Miocene birth of the Yangtze River. *Proc. Natl. Acad. Sci. U. S. A.* **110**, 7556–7561 (2013).
5. Deng, B., Chew, D., Jiang, L., Mark, C., Cogne, N., Wang, Z. J. & Liu, S. G. Heavy mineral analysis and detrital U-Pb ages of the intracontinental Palaeo-Yangtze basin: Implications for a transcontinental source-to-sink system during Late Cretaceous time. *Geol. Soc. Am. Bull.* **130**, 2087–2109 (2018).
6. Huang, H. Y., He, D. F., Li, D. & Li, Y. Q. Detrital zircon U-Pb ages of Palaeogene deposits in the southwestern Sichuan foreland basin, China: Constraints on

basin-mountain evolution along the southeastern margin of the Tibetan Plateau. *Geol. Soc. Am. Bull*. **130**, 2087–2109 (2020).

7. Sichuan Bureau of Geology and Mineral Resources. Regional Geology of Sichuan Province h.11&12 (part I): 264–282 (Geological Publishing House, 1991).
8. Gu, X. D. & Li, X. H. Lithostratigraphy in Sichuan Province 207–217, (China University of Geosciences Press, 1997).
9. Sichuan Bureau of Geology and Mineral Resources. Geological Map of the Qionglai Sheet (scale 1:200000), (Geological Publishing House Press, 1975).
10. Sichuan Bureau of Geology and Mineral Resources. Geological Map of the Emei Sheet (scale 1:50000), (Geological Publishing House Press, 1989).
11. Tang, Z. Biostratigraphic units in Mishi Basin in Panxi area. *Acta Geologica Sichuan.* 16, 209–212 (1996).
12. Sichuan Geological Survey Institute. The Report of Regional Geological Survey of Xichang at Scale 1: 250000, (China Industry Press, 2013).
13. Sichuan Bureau of Geology and Mineral Resources. Geological Map of the Xichang, Hexi, Xincun, and Guolianggai Sheet (scale 1:50000), (Geological Publishing House Press, 1990).
14. Carroll, A. R. & Bohacs, K. M. Stratigraphic classification ofancient lakes:balancing tectonic and climatic controls. *Geology* **27**, 99–102 (1999).
15. Bohacs, K. M., Carroll, A. R., Neal, J. E. & Mankiewicz , P. J. Lake-basin type, source potential, and hydrocarbon character: an integrated-sequence-stratigraphic–geochemical framework, in E. H. Gierlowski-Kordesch and K. R. Kelts, eds., Lake basins through space and time. *AAPG Stud. Geol.* **46**, 3–34 (2000).

16. Huang, R. J. Cretaceous to early Tertiary Charophytes in Sichuan Province. *Acta Micropalaeontologica Sinica* **2**, 77–89 (1985).
17. Jiang, Z. F., Cui, X. Z., Wu, H. & Zhuo, J. W. Detrital zircon LA-ICP-MS U-Pb ages of the Palaeogene Leidashu Formation in Huili, Sichuan Province and their geological significance. *Geological Bulletin of China* **33**, 788–803 (2014).
18. Yunnan Bureau of Geology and Mineral Resources. Geological Map of the Chuxiong Sheet (scale 1:200000), (Geological Publishing House Press, 1966).
19. Yunnan Bureau of Geology and Mineral Resources. Geological Map of the Chuxiong Sheet (scale 1:50000), (Geological Publishing House Press, 1996).
20. Yunnan Bureau of Geology and Mineral Resources. Geological Map of the Dayao Sheet (scale 1:200000), (Geological Publishing House Press, 1965).
21. Xue, C, D., Xiang, K., Hu, T. Y., Liao, C., Yang, X. P., Yang, T. Y. L. & Wang, L. Sedimentary Environments of Late Cretaceous Ore-bearing Sequences at the Guihua Copper Ore Field in the Northern Chuxiong Basin, Yunnan Province, SW China. *Acta Sedimentologica Sinica* **37**, 491–501 (2019).
22. Miall, A. D. (ed). Fluvial sedimentology. *Can Soc Petrol Geol Mem.* **5** (1978).
23. Saylor, J. E. & Sundell, K. E. Quantifying comparison of large detrital geochronology data sets. *Geosphere* **12**, 203–220 (2016).
24. Dickinson, W. R., Beard, S. L., Brakenridge, G. R., Erjavec, J. L., Ferguson, R. C., Inman, K. F., Knepp, R. A., Lindberg, F. A. & Ryberg, P. T. Provenance of North American Phanerozoic sandstones in relation to tectonic setting. *Geol. Soc. Am. Bull.* **94**, 222–235 (1983).

25. Ingersoll, R. V., Bullard, T. F., Ford, R. L., Grimm, J. P., Pickle, J. D. & Sares, S. W. The effect of grain size on detrital modes: A test of the Gazzi-Dickinson point-counting method. *J Sediment Petrol*. **54**, 103–116 (1984).
26. Chen, Y., Yan, M., Fang, X., Song, C., Zhang, W., Zan, J. et al. Detrital zircon U-Pb geochronological and sedimentological study of the Simao Basin, Yunnan: Implications for the early Cenozoic evolution of the Red River. *Earth Planet. Sci. Lett.* **476**, 22–33 (2017).
27. Li, Y. Q., He, D. F., Li, D., Lu, R. Q., Fan, C., Sun, Y. P. & Huang, H. Y. Sedimentary provenance constraints on the Jurassic to Cretaceous paleogeography of Sichuan Basin, SW China. *Gondwana Res.* **60**, 15–33 (2018).
28. Wang, L. C., Liu, C., Gao, X. & Zhang H. Provenance and paleogeography of the Late Cretaceous Mengyejing Formation, Simao Basin, southeastern Tibetan Plateau: Whole-rock geochemistry, U-Pb geochronology, and Hf isotopic constraints. *Sedimentary Geol*. **304**, 44–58 (2014).
29. Sundell, K. & Saylor, J.E. Unmixing detrital geochronology age distributions. *Geochem., Geophys., Geosyst*. **18**, 2872–2886 (2017).
30. Vermeesch, P. Multi-sample comparison of detrital age distributions. *Chem. Geol.* **341**, 140–146 (2013).
31. Wang, Y. L., Wang, L. C, Wei, Y. S., Shen, L. J., Chen, K., Yu, X. C. & Liu, C. L. Provenance and paleogeography of the Mesozoic strata in the Muang Xai basin, northern Laos: petrology, whole-rock geochemistry, and U–Pb geochronology constraints. *Int J Earth Sci*. **106**, 1409–1427 (2017).

32. Breitfeld, H. T. & Hall, R. The eastern Sundaland margin in the latest Cretaceous to Late Eocene: Sediment provenance and depositional setting of the Kuching and Sibu Zones of Borneo. *Gondwana Res.* **563**, 34–64 (2018).

33. Galin, T., Breitfeld, H. T., Hall, R. & Sevastjanova, I. Provenance of the Cretaceous–Eocene RajangGroup submarine fan,Sarawak,Malaysia from light and heavymineral assemblages and U-Pb zircon geochronology. *Gondwana Res.* **51**, 209–233 (2017).
