# Supplementary material for: Existence of a continental-scale river system in eastern Tibet during the late Cretaceous–early Palaeogene
Source: Nat Commun. 2021 Dec 13;12:7231. doi: 10.1038/s41467-021-27587-9 (PMC8668954; doi:10.1038/s41467-021-27587-9)
Supplement: Supplementary file 3 — Description of Additional Supplementary Files [file 41467_2021_27587_MOESM3_ESM.pdf]

Description of Additional Supplementary Files

File Name: Supplementary Data 1

Description: LA-ICP-MS zircon U-Pb data for all samples.

File Name: Supplementary Data 2

Description: Dating results of zircon standards.
